# Editorial: Ethical challenges in AI-enhanced military operations

**DOI:** 10.3389/fdata.2023.1229252

**Published:** 2023-06-19

**Authors:** Kirsi Marjaana Helkala, George Lucas, Edward Barrett, Henrik Syse

**Affiliations:** ^1^Norwegian Defence Cyber Academy, Norwegian Defence University College, Oslo, Norway; ^2^Peace Research Institute Oslo, Oslo, Norway; ^3^Department of Leadership, Ethics and Law, U.S. Naval Academy, Annapolis, MD, United States; ^4^Stockdale Center, U.S. Naval Academy, Annapolis, MD, United States

**Keywords:** artificial intelligence (AI), military ethics, virtue ethics, conventional combat, cyber conflict, strategic planning

This Research Topic primarily focuses on the people—military personnel throughout the command structure—who serve in combat settings with AI-enabled machines. In a battlespace where machine autonomy is increasingly assuming functions once restricted to human beings, maintaining clear lines of human responsibility is of paramount importance. Clarifying this issue should improve ethical instruction within military training and educational institutions, as well as change how AI developers design their technologies. In turn, this will render ethical guidelines better tailored to the battlefield scenarios military personnel will confront in the future.

This collection aims to yield moral guidelines for the variety of military uses of AI technology, primarily in three areas:

Conventional armed conflict/battlefield combat;
Cyber military operations and cyber conflict;
Strategic planning for war and data-driven battlefield management.

Additionally, these essays examine the impact on the competency and character of human operators, *inter alia*, through the lens of virtue ethics. That is, they focus on individual character and the cultivation of moral predispositions that empower us to act responsibly amid the challenges to personal and professional life increasingly posed by the use of artificial intelligence in cyber security, kinetic warfare, and intelligence and strategic planning.

Within cyber security, AI-based tools can be used in both offensive and defensive cyber applications, from malware detection, network intrusion, and phishing and spam detection to intelligent threats and tools for attacking AI models. AI-based systems and their use in the kinetic battlefield encompass autonomous vehicles, drones, and swarms. Finally, intelligence systems that enable the examination and analysis of large data sets and integration of inputs from a vast array of sources enable ever-more effective planning, battlefield management, surveillance, and development of data-driven strategies focused on defense and national security (as is currently happening in Ukraine).

This Research Topic of “Frontiers in Big Data” originated as part of a project (“Warring with Machines: Artificial Intelligence and the Relevance of Virtue Ethics”) at the Peace Research Institute Oslo (PRIO) focused on the uses and ethical impact of AI in military settings and special operations. Most of the papers were refereed and presented initially at international conferences held in Rome (and co-organized by PRIO and Notre Dame University's Technology Ethics Center), at the McCain Conference in Annapolis, USA (organized by the Stockdale Center at the US Naval Academy in association with PRIO), as well as at annual conferences of the International Society of Military Ethics in Europe and the USA. Others were received in response to an international CFP through the Loop Science Network.

The papers encompass four research areas, each addressing the challenges of AI-enhanced military operations: (1) Artificial Intelligence and the Ethics of Warfare; (2) The Impact of Military Reliance on AI upon Human War Fighters and Their Control of Warfare; (3) Dignity and Respect; and (4) Gender Bias in Narratives of War.

The first topic includes a paper published separately from this Research Topic by Frontiers, namely, Regan and Davidovic (2023) “Just Preparation for War and AI-Enabled Weapons”, as well as the papers “*The Comparative Ethics of Artificial-intelligence Methods for Military Applications*” by Rowe, and “*The PRC Considers Military AI Ethics: Can Autonomy be Trusted?*” by Metcalf. All three address AI in warfare from a military ethics perspective, addressing proper preparation and testing, the ethics of different algorithms in use, and the deeply political way in which the military ethics of AI is understood in China.

The second topic is addressed by Hovd in the paper “*Tools of War and Virtue-Institutional Structures as a Source of Ethical Deskilling*”, which analyses military virtues as a species of moral virtues mediated by institutional and technological structures, meaning that professional roles and institutional structures are constitutive parts of what makes these virtues what they are, and thus the most likely source of ethical deskilling. The paper “*On the Purpose of Meaningful Human Control*” by Davidovic critically discusses and analyses calls for proper control of AI-enabled weapons systems, focusing on the *purpose* of such control, while “*The Ethics of AI-assisted War Fighter Enhancement Research and Experimentation*” by Moreno et al. discusses the problem of AI-wired war fighters facing affronts to cognitive liberty and to psychological and physiological health, as well as obstacles to integrating into military and civil society during their service and upon discharge, emphasizing the importance of ethics in the research underlying the use of such technologies.

The third topic includes the paper “*Resolving Responsibility Gaps for Lethal Autonomous Weapon Systems*” by Taylor Smith, in which the author suggests an understanding of collective responsibility for AI outcomes that can help resolve the “problem of many hands” and “responsibility gaps”. It also includes Kahn's “*Lethal Autonomous Weapons Systems and Respect for Human Dignity*”, which discusses whether actions involving the use of AI-enhanced weapons can respect human dignity. Kahn suggests criteria for the possibility of answering that question in the affirmative. Finally, “*Lethal Autonomous Weapons Systems, Revulsion, and Respect*” by Dean raises the issue of respect for public opinion and conventional attitudes as one develops lethal autonomous weapons systems, those opinions and attitudes often being very skeptical toward the development of such weaponry.

The fourth and last topic is addressed by Fisher in “*The Role of Gender in Providing Expert Advice on Cyber Conflict and Artificial Intelligence to Military Personnel*”. Fisher argues that, as the role of cyber and AI grows in military operations, there is a need for military institutions to take gender into account in both training and policy, not least due to the fact that gender stereotypes attach to the role of cyber-engineer.

Together, these papers focus on the impact of current and anticipated military uses of AI-augmented technologies within the framework of military ethics and the just war tradition, as well as unique individual moral challenges that such technologies present. The effect of AI-augmentation on conceptions of human dignity, respect, and gender roles in military settings is found to be particularly problematic. Specific responses and remedies to the major problems described are proposed where feasible.

## Author contributions

Each contributing author to this collection participated in conceptualization of their topic, writing of the original draft and subsequent revisions, and funding acquisition for the overall project. The editors approved the final submitted version.
